# Fibrin monomers evaluation during hospitalization for COVID-19 is a predictive marker of in-hospital mortality

**DOI:** 10.3389/fcvm.2023.1001530

**Published:** 2023-03-30

**Authors:** David M. Smadja, Nicolas Gendron, Aurélien Philippe, Jean-Luc Diehl, Nadège Ochat, Olivier Bory, Agathe Beauvais, Alexis Mareau, Anne-Sophie Jannot, Richard Chocron

**Affiliations:** ^1^ Innovative Therapies in Hemostasis, INSERM, University Paris Cité, Paris, France; ^2^ Hematology Department, AP-HP.CUP, Georges Pompidou European Hospital, Paris, France; ^3^ Intensive Care Unit, AP-HP.CUP, Georges Pompidou European Hospital, Paris, France; ^4^ Emergency Department, AP-HP.CUP, Georges Pompidou European Hospital, Paris, France; ^5^ Centre de Recherche des Cordeliers, AP-HP.CUP, Georges Pompidou European Hospital, Medical Informatics, Biostatistics and Public Health Department, European Georges Pompidou Hospital, AP-HP.CUP, University Paris Cité, Paris, France; ^6^ PARCC, INSERM, University Paris Cité, Paris, France

**Keywords:** SARS-CoV-2, COVID-19, fibrin monomers, microthrombosis, coagulopathy, ICU, mortality, D-dimer

## Abstract

**Background:**

Coagulopathy is one of the main triggers of severity and worsening of Coronavirus disease 2019 (COVID-19) particularly in critically ill patients. D-dimer has been widely used to detect COVID-19 coagulation disorders and has been correlated with outcomes such as disease severity and in-hospital mortality. Involvement of other fibrin degradation products, particularly fibrin monomers (FM), remains an ongoing question.

**Methods:**

We performed a monocentric study of adult patients with COVID-19, who were admitted either in the medical ward (MW) or in the intensive care unit (ICU) and who had FM measurements performed on them during the first wave of COVID-19 outbreak. We analyzed the positivity of FM levels (FM > 7 µg/mL) to assess the ability of FM monitoring during the first days of hospitalization to predict COVID-19 outcomes.

**Results:**

In our cohort, 935 FM measurements were performed in 246 patients during their first 9 days of hospitalization. During patient follow-up, the FM levels were higher in patients admitted directly to the ICU than in those admitted to the MW. Moreover, we observed significantly increased levels of FM in patients when the data were stratified for in-hospital mortality. At hospital admission, only 27 (11%) patients displayed a positive value for FM; this subgroup did not differ from other patients in terms of severity (indicated by ICU referral at admission) or in-hospital mortality. When analyzing FM positivity in the first 9 days of hospitalization, we found that 37% of patients had positive FM at least once during hospitalization and these patients had increased in-hospital mortality (*p* = 0.001). Thus, we used non-adjusted Kaplan–Meier curves for in-hospital mortality according to FM positivity during hospitalization and we observed a statistically significant difference for in-hospital mortality (hazard ratio = 1.48, 95% CI: 1.25–1.76, *p* < 0.001). However, we compared the AUC of FM positivity associated with a ratio of D-dimer >70% and found that this combined receiver operating characteristic (ROC) curve was superior to the FM positivity ROC curve alone.

**Conclusion:**

Monitoring of FM positivity in hospitalized patients with COVID-19 could be a reliable and helpful tool to predict the worsening condition and mortality of COVID-19.

## Introduction

Coagulopathy is one of the main triggers of disease severity and fatal outcome in Coronavirus disease 2019 (COVID-19), particularly in critically ill patients (1, 2). Inflammation and related endothelial lesions are probably at the origin of this coagulopathy associated with pulmonary vascular obstruction (2). Coagulation activation during COVID-19 is largely reflected by an increase in D-dimer levels (3). Extensive literature exists on the predictive value of D-dimer (4), mainly at patient admission in hospital, and D-dimer measurement in the first 48 h after admission has been proposed as a sensitive biomarker of initial severity and in-hospital mortality (5). In our previous multicenter study involving 24 French hospitals, 1,154 patients had D-dimer measurement performed on them upon admission in the medical ward (MW). We demonstrated that a D-dimer level above 1,128 ng/mL was a relevant predictive factor for in-hospital mortality among patients admitted in the MW for COVID-19. This held true regardless of the occurrence of venous thromboembolism (VTE) during hospitalization (6). More recently, we proposed a new algorithm with a specific D-dimer threshold in COVID-19 patients according to lung extension disease, to safely exclude pulmonary embolism (PE) (7) and to reduce the use of the computed tomography pulmonary angiogram. Finally, we demonstrated that daily monitoring of D-dimer was a hallmark of severe COVID-19 disease (8), since modified kinetics are associated with intensive care unit (ICU) referral and in-hospital mortality.

The coagulation process results in an insoluble clot of cross-linked fibrin. Subsequently, the fibrinolytic system activates to limit the clot size. Lastly, plasmin degrades cross-linked fibrin into different soluble fragments, including D-dimer. Disseminated intravascular coagulation (DIC) was first suspected in SARS-CoV-2 infection in early reports of COVID-19. DIC is characterized by the systemic activation of coagulation, which can lead either to thrombosis of the small and midsize vessels, contributing to organ failure, or to bleeding with platelet and coagulation factor consumption. DIC is secondary to other conditions such as severe infection, cancer, trauma, or obstetric complications. The International Society on Thrombosis and Haemostasis (ISTH) established a scoring algorithm for the diagnosis of DIC. The score relies on platelet count, prolonged prothrombin time, fibrinogen level (9), and the level of fibrin markers, including D-dimer. Nevertheless, D-dimer is not a specific biomarker; it also increases in pregnancy, inflammatory disease, and sepsis, for instance. The results must therefore be interpreted along with clinical features and other laboratory assays. The loss of coagulation factors or antithrombin and acquisition of a DIC-like phenotype was not typically seen in COVID-19 (10). However, this does not prevent the patient from developing DIC, although this is a rather rare event in COVID-19 until the later stages of severe disease (1). Another fibrin-related biomarker is fibrin monomers (FM) whose concentration has been largely described to reflect prothrombin activity. FM have been proposed as a diagnostic marker of DIC (9) and a predictor of thrombosis and/or a hypercoagulable state earlier than D-dimer (11). In healthy individuals, FM levels are very low in peripheral blood, generally below the limit of detection (12). In the early stages of the pandemic, some experts proposed evaluating FM levels in COVID-19 patients (13). Despite these expert recommendations, less data are available on the relevance of FM evaluation during COVID-19 (14–19) in contrast to D-dimer. None of the studies made a strong case for a clear-cut clinical use and a daily-life evaluation.

The aim of the present study was to determine, with a large retrospective study, the incidence of FM positivity among adult patients hospitalized for COVID-19 both at admission and daily during hospitalization. In addition, we examined the prognostic value of FM positivity when assessing in-hospital mortality.

## Methods

### Study design and population

We performed a monocentric study of adult patients (≥18-years old) with COVID-19, who were admitted in the European Georges Pompidou Hospital between 1 February and 30 June 2020. We retrospectively analyzed, for the study period, all patients with a confirmed diagnosis of COVID-19, using a reverse transcriptase–polymerase chain reaction as previously described (8, 20), and who had FM measurements performed on them. The patients were classified according to World Health Organization (WHO) guidelines as either non-critical (median oxygen requirement of 3 L/min and a WHO score range of 4–7) or critical (requiring mechanical ventilation, WHO score range 8–9). Patient characteristics included age, sex, and body mass index (BMI). Clinicians used the local protocol recommended during this period. This protocol was based on the international guidelines from the ISTH, advocating for the use of a prophylactic regimen of anticoagulation for both non-critical patients and critical patients admitted in the ICU. Few patients had therapeutic or intermediate prophylaxis dose in surgical intensive care. No patient received glucocorticoids or immunosuppressant treatments. Venous blood was collected from patients in 0.129 M trisodium citrate tubes (9NC BD Vacutainer, Plymouth, UK) and processed according to standard laboratory techniques. Platelet-poor plasma was obtained after centrifugation twice at 2,500× *g* for 15 min at room temperature.

**Figure 1:**
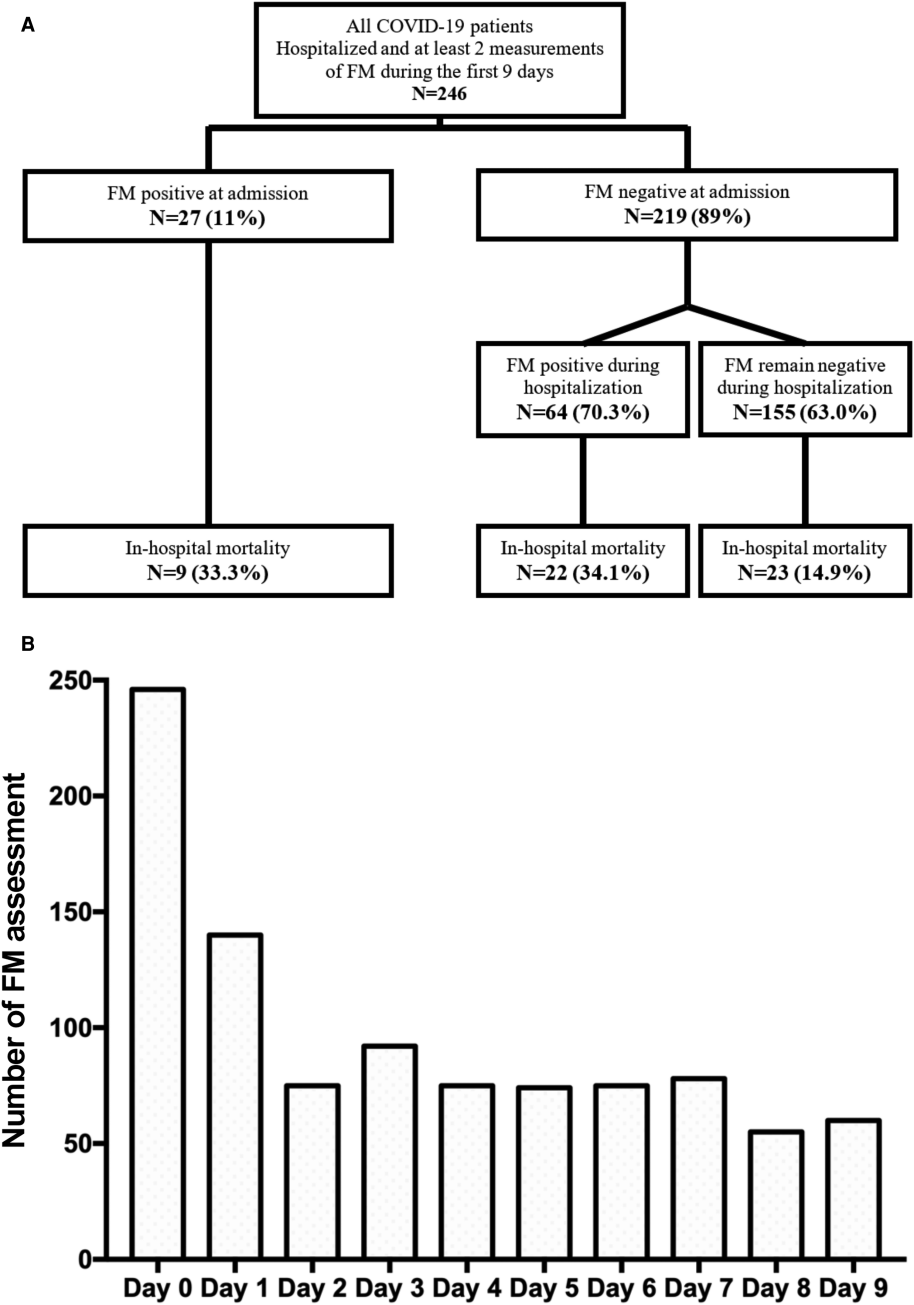
Population description. (**A**) Study flow chart. We solely included patients who had been hospitalized and whose FM levels had been measured at least twice during the first 9 days of hospitalization. We excluded all patients who did not meet these criteria. (**B**) Number of FM level assessments.

We studied only those patients who were hospitalized and whose FM levels had been measured at least twice during the first 9 days. We excluded all patients who did not meet these criteria. The flow chart of our study is presented in Figure 1A. The median [interquartile range (IQR)] number of FM tests was 3 [2–6], with a minimum of 2 and a maximum of 10 (Day 0 for day of admission; Figure 1B).

### Fibrin monomer evaluation

We quantified the plasma FM level in platelet-poor plasma (STA-Liatest FM, Diagnostica Stago, France) using a STA-R Max analyzer (Diagnostica Stago, as recommended by the manufacturer). According to the manufacturer's recommendation and calibration curves, the limit of detection was 6 µg/mL. Furthermore, as part of a requirement of the COFRAC ISO 15189 label of our laboratory, the limit of quantification was determined in our coagulometers and was identified as 7 µg/mL. Hence, positivity for FM was indicated by values above 7 µg/mL. The local protocol for COVID-19 management suggested the physician to measure FM in COVID-19 patients at admission and thereafter 72 h.

### Statistical analysis

For the descriptive analysis, data were expressed as the median (IQR) for continuous data and as frequencies and percentages for categorical data. We used the Mann–Whitney test and Fisher's exact test to compare the continuous and categorical variables, respectively. In the survival analysis, we used the Kaplan–Meier curve to estimate the survival function from diagnosis to in-hospital death stratified to FM positivity. Survival curves were compared using the log rank test. We used the Cox proportional hazard (PH) model adjusted for age, BMI, and sex to investigate the association between in-hospital mortality and FM positivity. FM was a binary variable coded as follows: If the patient had at least one positive measurement above 7 µg/mL during the first 9 days, FM positivity was coded as 1; if the patient had any other positive measurement with FM ≤ 7 µg/mL, FM was coded as 0. To assess the PH model assumptions, we first estimated the regression coefficients using the functions *coxph* (*package survival*) and *crr* (*package cmprsk*). We tested for PH using the functions *prop.coxph* and *prop.crr* with default resampling methods implemented by the *goftte* package. We used two methods to examine the changes in the value of FM over time within the patient groups, which are defined as follows: (1) ICU at admission, (2) MW at admission and then transferred to the ICU during hospitalization, and (3) MW during the whole period of hospitalization. In the first method, during the first 9 days of hospitalization, we compared the mean value of FM in the group of patients who were admitted in the ICU with the mean value of patients admitted in an MW, using a Wilcoxon test with Bonferroni-corrected alpha level applied for each comparison. Second, to assess how the value of FM changed over time within each of the three patient groups, we used a linear mixed-effect model. In the model, time was considered a continuous measure, the patient group variable was an independent variable, and we added a random effect for the patient and an interaction term between the patient group and the period of time. To assess the prognostic ability of FM positivity and D-dimer value—at admission and during follow-up—we used receiver operating characteristic (ROC) curve analysis and compared the results with DeLong's test. The issue of missing data were addressed by imputation using a linear interpolation from observed values with an approximation function of the *stats* package of R software. Based on the imputed data, we created a graph using the *geom_smooth* function in the *ggplot2* visualization package of R. All analyses were two-sided, and statistical significance was set to *p* < 0.05. Statistical analyses were performed using R studio software, including R version 3.6.3 (RStudio Inc., Boston, MA, USA).

## Results

### FM levels during the first 9 days of hospitalization differed according to clinical outcomes in COVID-19 patients

**Figure 2:**
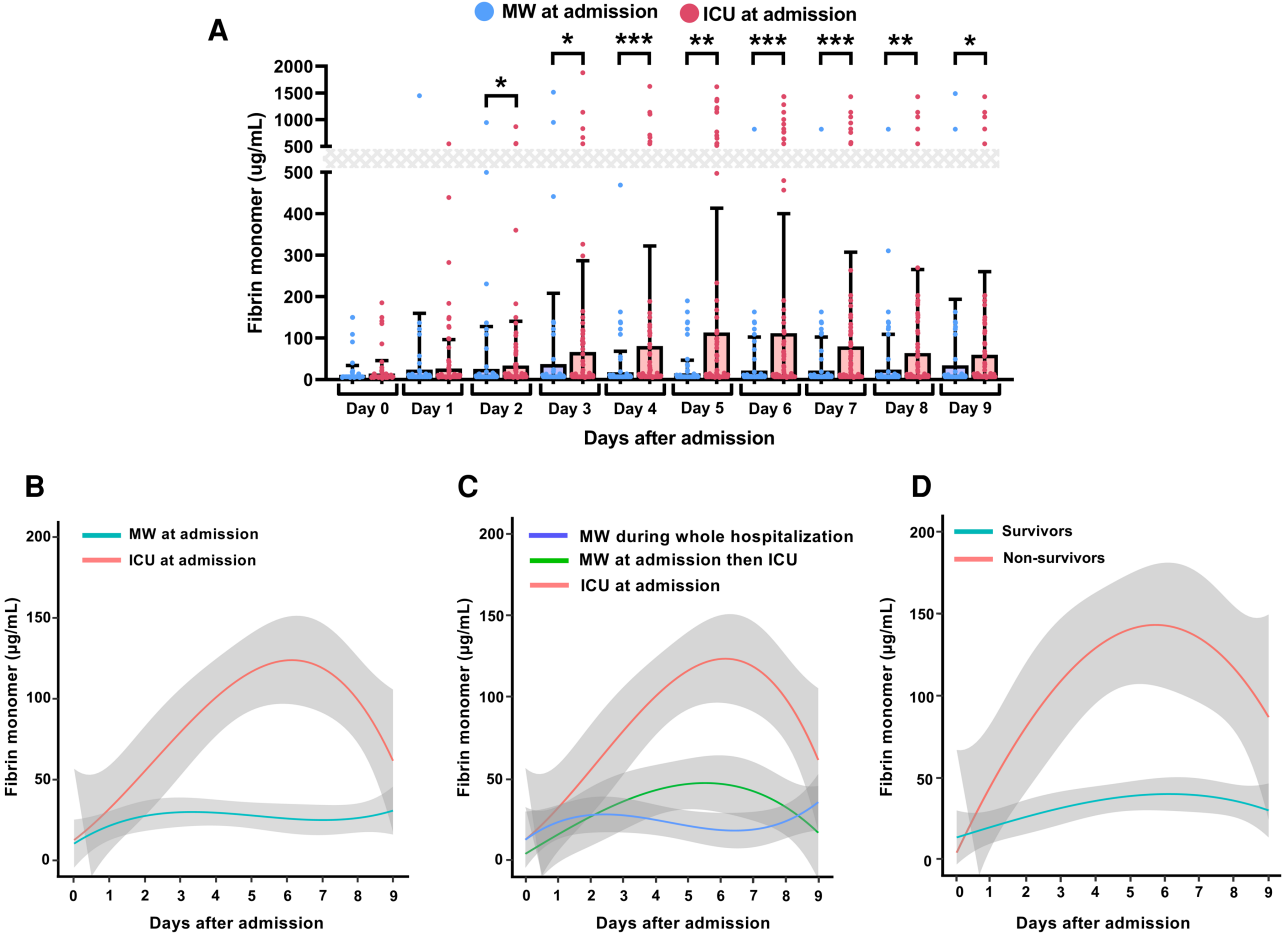
Daily monitoring of fibrin monomers levels and outcome predictions in hospitalized COVID-19 patients. For each group, the lines indicate the trendline of the mean FM levels measured daily during the first 9 days of hospitalization and the gray-colored area denotes the 95% confidence interval. *p ≤ 0.05, **p ≤ 0.01, ***p ≤ 0.001. (**A**) Comparison of FM levels during the first 9 days of hospitalization. (**B**) Temporal trends of daily FM levels during the first 9 days of hospitalization according to initial admission at hospital: medical ward (MW; blue Line) or intensive care unit (ICU; red line). (**C**) Temporal trends of daily FM levels during the first 9 days of hospitalization according to the care pathway: MW during the whole hospitalization (blue line); MW at admission then referred to the ICU (green line); ICU at admission (red line). (**D**) Temporal trends of daily FM levels during the first 9 days of hospitalization according to in-hospital mortality: survivors (blue line); non-survivors (red line).

Overall, 935 FM measurements were performed for 246 patients during their first 9 days of hospitalization (Figure 1B). Since the number of measurements of FM after 9 days of hospitalization was very low, we restricted our analysis because of low statistical power after more than 9 days. In our cohort, 169 (68.7%) patients were male, the median age was 66.0 years (IQR: 56.0–76.0), and 28 patients (11.4%) had a BMI above 30 kg/m^2^. The cohort included 154 (62.6%) patients with COVID-19 who were initially admitted in an MW and 92 (37.4%) initially admitted in the ICU. The median duration of hospital stay was 16.0 days (IQR: 7.0–25.5) and the median delay from hospital admission to in-hospital death was 12.5 days (IQR: 6.0–24.0). The study period corresponded to the first wave of the pandemic, and clinicians used the local protocol recommended during this period. This protocol was based on the international guidelines from the ISTH and included the use of a prophylactic regimen of anticoagulation for non-critical patients and critical patients admitted in the ICU. A few patients underwent therapeutic or intermediate prophylactic dose in the surgical ICU (13, 21). None of the patients received glucocorticoids/immunosuppressants treatments. Table 1 describes the range and positivity of FM observed during the first 9 days of hospitalization. In comparison with COVID-19 patients admitted in the MW, the mean FM levels of patients directly admitted in the ICU were not different at Day 0 and Day 1 (Figure 2A). In contrast, from Day 3 onwards in the MW, the mean FM levels were significantly higher in patients admitted initially in the ICU than in those admitted initially in the MW (*p* < 0.05, Wilcoxon test for all days following admission). Consistently, by considering the trend in FM levels throughout the period of the patients' follow-up, we found that the mean FM levels were significantly higher in patients admitted directly in the ICU than in those admitted to the MW (Figure 2B). Among the 154 COVID-19 patients directly admitted to the MW, 40 (26%) were referred to the ICU during their hospitalization, whereas 114 (74%) remained in the MW. Among patients initially admitted in the MW, starting from Day 4, a significant increase in FM levels was evidenced only for those referred to the ICU (*p* < 0.001, Wilcoxon test; Figure 2C). Finally, we observed significantly increased levels in the mean FM levels in patients (Figure 2D) when stratified on survival.

**Table 1:** Patients’ value of fibrin monomers during hospitalization for COVID-19.

		All population		ICU at admission		MW at admission and then ICU		MD during the whole period of hospitalization	
		*n* = 246		*n* = 92		*n* = 40		*n* = 114	
Days	FM positivity	FM		FM		FM		FM	
	*n* (%)	Median [IQR]	Mean (SD)	Median [IQR]	Mean (SD)	Median [IQR]	Mean (SD)	Median [IQR]	Mean (SD)
0	27 (10.9%)	7.0 [7.0–7.0]	13.4 (27.2)	7.0 [7.0–7.0]	18.1 (36.4)	7.0 [7.0–7.0]	7.6 (3.2)	7.0 [7.0–7.0]	11.7 (22.5)
1	49 (19.9%)	7.0 [7.0–7.0]	25.4 (105.6)	7.0 [7.0–7.3]	31.9 (81.5)	7.0 [7.0–7.0]	14.3 (28.7)	7.0 [7.0–7.0]	24.1 (136.0)
2	48 (19.5%)	7.0 [7.0–7.0]	30.2 (104.5)	7.0 [7.0–8.8]	39.9 (122.0)	7.0 [7.0–7.7]	20.4 (56.8)	7.0 [7.0–7.0]	25.7 (102.2)
3	51 (20.7%)	7.0 [7.0–7.0]	52.9 (199.2)	7.0 [7.0–10.6]	80.7 (256.8)	7.0 [7.0–9.6]	33.1 (87.8)	7.0 [7.0–7.0]	37.3 (171.0)
4	57 (23.2%)	7.0 [7.0–7.0]	51.7 (182.6)	7.0 [7.0–14.9]	98.2 (277.6)	7.0 [7.0–19.0]	41.6 (117.1)	7.0 [7.0–7.0]	17.7 (50.4)
5	59 (24.9%)	7.0 [7.0–7.0]	67.9 (226.0)	7.0 [7.0–26.2]	134.3 (329.5)	7.0 [7.0–13.9]	64.5 (215.2)	7.0 [7.0–7.0]	15.5 (31.4)
6	61 (25.8%)	7.0 [7.0–7.8]	70.3 (222.2)	7.0 [7.0–51.1]	139.0 (325.8)	7.0 [7.0–9.1]	50.2 (158.4)	7.0 [7.0–7.0]	21.8 (80.6)
7	58 (24.6%)	7.0 [7.0–7.0]	53.2 (177.2)	7.0 [7.0–35.9]	102.4 (266.7)	7.0 [7.0–8.0]	29.2 (56.0)	7.0 [7.0–7.0]	21.9 (80.8)
8	54 (22.9%)	7.0 [7.0–7.0]	45.5 (159.5)	7.0 [7.0–13.2]	80.1 (237.7)	7.0 [7.0–7.1]	26.8 (49.6)	7.0 [7.0–7.0]	24.1 (85.2)
9	50 (20.3%)	7.0 [7.0–7.0]	48.1 (182.6)	7.0 [7.0–10.5]	75.8 (237.0)	7.0 [7.0–7.0]	23.5 (43.8)	7.0 [7.0–7.0]	34.4 (159.4)

ICU, intensive care unit; MW, medical ward; FM, fibrin monomers; IQR, interquartile range; SD, standard deviation.

### Iterative FM measurements during hospitalization, but not FM levels at admission, were predictive of worsening COVID-19 and in-hospital mortality

**Figure 3:**
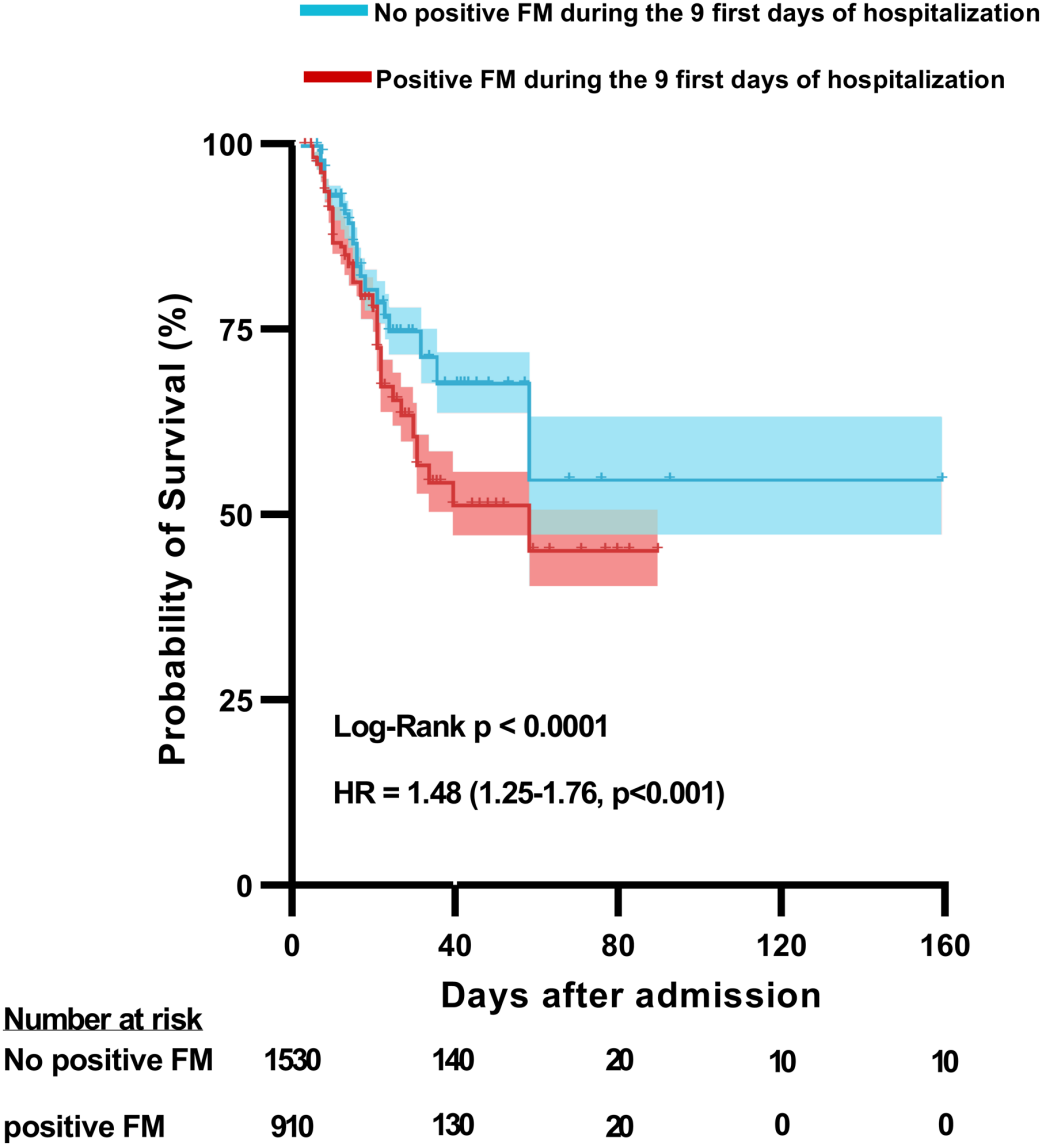
In-hospital mortality according to fibrin monomer positivity during the first 9 days of hospitalization for COVID-19. Unadjusted Kaplan–Meier survival curve for mortality among all hospitalized COVID-19 patients according to whether patients displayed at least one positive fibrin monomer (FM) level (Blue line) or not (Red line) during hospitalization. The *p*-value refers to the log rank test. Estimates of the hazard ratio (HR) and 95% confidence intervals derived from a single Cox-model model assessing the association between at least one positive FM level during hospitalization and in-hospital mortality.

At hospital admission, only 27 (11%) patients displayed a positive value of FM (FM > 7 µg/mL, 17 of these patients were admitted in the ICU and 10 in MW). We analyzed the positivity of FM levels (FM > 7 μg/mL) to assess the ability of FM monitoring during the first days of hospitalization to predict COVID-19 outcomes. We first analyzed FM positivity at hospital admission (Day 0). As demonstrated in Table 2, patients having positive FM at admission showed no differences in terms of age, sex, and BMI and no difference was evident for severity (ICU referral at admission) or in-hospital mortality. Thus, we demonstrate that evaluating FM specifically at admission has no significance in terms of severity or in-hospital mortality prediction. In contrast, an analysis of FM positivity during the first 9 days of hospitalization indicated that 155 (63%) patients had negative FM during the whole period of hospitalization, whereas 91 (37%) patients had positive FM at least once during their stay in hospital. Patients having at least one positive FM during hospitalization did not show any differences in terms of age, sex, and BMI. However, they required more ICU referral at admission (*p* < 0.001) and had increased in-hospital mortality (*p* = 0.001). To confirm the association between FM positivity during the first 9 days of hospitalization and in-hospital mortality, we generated a non-adjusted Kaplan–Meier curve of in-hospital survival (Figure 3). The result demonstrated a statistical difference [hazard ratio (HR) 1.48, 95% CI: 1.25–1.76, *p* < 0.001]. Finally, a Cox regression model for in-hospital mortality adjusted for age, sex, and BMI confirmed a significant difference for in-hospital mortality of patients with positive FM during the first 9 days of hospitalization (adjusted HR: 1.47, 95% CI: 1.23–1.76, *p* = 0.001, data not given). To complete our analysis, we conducted a logistic regression test to determine whether the positivity of FM was associated with ICU referral. The results confirmed the link between FM positivity and ICU referral with an odds ratio of 2.27 (95% CI: 1.65–3.12, *p* < 0.001, data not shown). Finally, among patients with at least one positive FM during hospitalization, we analyzed the difference between those patients with positive FM at admission (Day 0) and those with positive FM solely during hospitalization (Day 1 in MW). We observed no significant differences in terms of demographics, COVID-19 severity, or in-hospital mortality.

**Table 2:** Patients’ characteristics and outcomes according to fibrin monomer measurement during hospitalization for COVID-19.

		Negative	Positive	*p*-value
FM measured at admission in hospital (*n* = 246)				
Patients, *n* (%)		219 (89.0)	27 (11.0)	
Male, *n* (%)		151 (68.9)	18 (66.7)	0.98
Age, years [median (IQR)]		66.0 [55.5–76.0]	65.0 [59.5, 77.0]	0.97
BMI ≥ 30 kg/m^2^, *n* (%)		26 (11.9)	2 (7.4)	0.71
Median [IQR]		7.0 [7.0–7.0]	29.0 [16.5–134.5]	<0.001
ICU admission (%)	ICU at admission	115 (52.5)	17 (63.0)	0.41
	Medical ward at admission	104 (47.5)	10 (37.0)	
In-hospital mortality, *n* (%)		45 (20.5)	9 (33.3)	0.21
		Negative	Positive	*p*-value
FM measured during the whole period of hospitalization (*n* = 246)				
Patients, *n* (%)		155 (63.0)	91 (37.0)	
Male, *n* (%)		105 (67.7)	64 (70.3)	0.78
Age, years, median [IQR]		66.0 [55.0–75.0]	66.0 [58.0–78.0]	0.53
BMI ≥ 30 kg/m^2^, *n* (%)		22 (14.2)	6 (6.6)	0.11
Median [IQR]		7.0 [7.0–7.0]	11.0 [7.0–88.9]	<0.001
FM at admission in hospital	ICU at admission	46 (29.7)	46 (50.5)	<0.001
	Medical ward at admission	109 (70.3)	45 (49.5)	
In-hospital mortality, *n* (%)		23 (14.9)	31 (34.1)	0.001
		Positive during follow-up	Positive at admission only	
Positive FM measured at least one time during hospitalization (*n* = 91)				
Patients, *n* (%)		64 (70.3)	27 (29.7)	
Male (%)		46 (70.4)	18 (66.7)	0.83
Age, years [median (IQR)]		66.0 [58.0–78.0]	65.0 [59.5–77.0]	0.78
BMI ≥ 30 kg/m^2^, *n* (%)		4 (6.6)	2 (7.4)	1.00
Median [IQR]		10.0 [7.0–85.0]	29.0 [16.5–134.5]	0.001
FM at admission in hospital	ICU at admission	29 (70.6)	17 (63.0)	0.52
	Medical ward at admission	35 (29.4)	10 (37.0)	
In-hospital mortality, *n* (%)		22 (34.1)	9 (33.3)	1

ICU, intensive care unit; MW, medical ward; FM, fibrin monomers; IQR, interquartile range; SD, standard deviation.

### Combination of FM positivity and the ratio of D-dimer assessed during the first 9 days of hospitalization improves in-hospital mortality prediction

We previously described a ratio of D-dimer (RoD) defined as either the D-dimer value on the day of outcome occurrence or the highest value during the first 9 days (if the outcome did not occur), divided by the D-dimer level at admission (8). We also previously identified different optimal thresholds for RoD: for patients with COVID-19 admitted directly to the MW, a threshold of 69% increase in RoD predicted in-hospital mortality; for patients admitted directly to the ICU, a threshold of 74% increase in RoD predicted in-hospital mortality (8). Thus, we compared the RoD predictive value with FM positivity and D-dimer at admission or FM positivity and RoD > 70% during hospitalization. An ROC curve analysis was created using D-dimer levels, FM positivity, and both parameters together at admission for the prediction of in-hospital mortality. In addition, we performed an ROC curve analysis for RoD > 70%, FM positivity, and both parameters together during hospitalization for the prediction of in-hospital mortality. We compare the predictability of three ROC curves in Table 3 with DeLong's test for two correlated ROC curves 2 by 2 using the package *pROC*. No significant difference was found at admission regarding the ability to predict in-hospital mortality among positive FM, D-dimer, or both criteria. However, we compared the AUC of FM positivity associated with an RoD > 70% and found that this combined ROC curve was superior to the FM positivity ROC curve alone. This result demonstrates that FM is not superior to RoD > 70% during hospitalization, but that adding positivity to ROD allows us to more accurately predict in-hospital mortality.

**Table 3:** D-dimer and FM involvement during hospitalization for COVID-19: comparison of ROC curve analysis associating D-dimer or FM at admission or during the first 9 days of hospitalization.

Delong's test for two correlated ROC curves 2 by 2	ROC curve for FM positivity at admission	ROC curve for FM positivity and D-dimer at admission
ROC curve for FM positivity at admission	–	*p* = 0.66
ROC curve for D-dimer at admission	*p* = 0.65	*p* = 0.63
	ROC curve for FM positivity during hospitalization	ROC curve for FM positivity and RoD > 75% during hospitalization
ROC curve for FM positivity during hospitalization	–	*p = 0.0009
ROC curve for RoD > 70% during hospitalization	*p* = 0.09	*p* = 0.63

## Discussion

Coagulation activation is a hallmark of COVID-19 severity and probably reflects microthrombosis. Endotheliopathy associated with SARS-CoV-2 infection could be explained at least in part by coagulopathy, microthrombosis, and severity of symptoms (2). In this study, we demonstrated that FM positivity measured during the first 9 days of hospitalization was associated with COVID-19 severity and in-hospital mortality. Using a monocentric study of patients hospitalized for COVID-19, we observed that patients who displayed positive FM had a higher in-hospital mortality rate than those who never displayed a positive FM measurement during the first 9 days of hospitalization. Importantly, our study explored the usefulness of FM in a large cohort of patients with COVID-19 of varying degrees of clinical severity.

In terms of biomarkers, hemostasis parameters such as D-dimer have been highly “popular” and relevant biomarkers during the COVID-19 outbreak. Thrombus formation could be the origin of increased D-dimer observed in COVID-19; however, D-dimer can also originate from the extravascular space through the breakdown of alveolar fibrin deposits (22, 23). This extravascular origin could explain why FM plasma levels are generally within the normal range but D-dimer is high (16). Indeed, D-dimer is the most frequently observed abnormal coagulation parameter measured during SARS-CoV-2 infection. Before the COVID-19 outbreak, D-dimer was used in clinical practice to exclude a diagnosis of VTE, to estimate the risk of VTE recurrence, and to support the diagnosis of DIC (3, 9). The D-dimer levels at admission is an important indicator of COVID-19 severity and a relatively accurate good predictor of a worsening clinical state and in-hospital mortality, independent of VTE risk (6). Moreover, the course of D-dimer levels during hospitalization also seems relevant and may better predict outcomes (8). Thus, in contrast to early descriptions, it is now clear that increased D-dimer and COVID-19-induced coagulopathy is a clinical entity that differs from DIC, at least in part, at admission.

D-dimer is a specific marker of fibrin degradation (24), but it is not specific to VTE or DIC. Many conditions and diseases may increase D-dimer levels, including pregnancy and inflammatory diseases. The D-dimer/fibrinogen ratio has been proposed to increase the specificity of VTE diagnosis, but the results are inconsistent (25). With the high involvement of coagulopathy in COVID-19 for mortality prediction, it is important to identify other fibrin degradation biomarkers such as FM. FM is produced by thrombin cleavage of fibrinogen, releasing fibrinopeptide A and B, and has been proposed as a marker of DIC or thrombosis (12). The presence of FM in circulation indicates an ongoing process of fibrinogen transitioning to fibrin after thrombin activation. In COVID-19, FM evaluation has been proposed by the French Society of Anesthesiology in the event of clinical worsening (13). However, little data on FM evaluation are available. Godon et al. studied a cohort of 164 COVID-19 patients and found that FM did not add any benefit, in contrast to D-dimer, to predict thrombotic events (14); analyzed events included VTE, arterial thrombosis, catheter-related thrombosis, and clotting related to dialysis filter and extracorporeal membranous oxygenation (14). Sridharan et al. demonstrated that only 23% of COVID-19 patients with increased D-dimer levels had elevated FM (15). This result is interesting in terms of specificity for DIC or COVID-19-associated coagulopathy diagnoses.

We previously described the clinical interest of FM monitoring for patients who are supported with ventricular assist device in order to assess appropriate anticoagulation (26, 27). Indeed, these patients had high levels of D-dimer, without any detectable thrombotic events, but interestingly, D-dimer levels were correlated to the amount of fibrin deposits on the surface of hemocompatible materials. In these patients, due to their high basal D-dimer level, DIC secondary to cessation of anticoagulant therapy is not easy to diagnose using D-dimer level alone, in contrast to FM (26, 27). In patients with COVID-19, FM levels are generally below the limit of detection and hence negative, whereas D-dimer is elevated in most of these patients and particularly those admitted in the ICU (8). Furthermore, the interpretation of D-dimer results in large cohorts may involve some challenges because of different reagents used in healthcare laboratories as stated by ISTH SSC during the COVID-19 outbreak (28, 29). Indeed, D-dimer assays can yield non-identical results. The reasons are differences in antibody specificity because of the heterogeneity in affinity for high- or low-molecular-weight fibrin degradation products and/or cross-linked and non-cross-linked fibrin derivatives.

Here, we demonstrate that evaluating FM specifically at admission has no relevance in terms of severity stratification or in-hospital mortality prediction. However, FM positivity during the follow-up period and its capacity to predict in-hospital mortality in any period are could be of clinical interest. D-dimer is associated with fibrin deposits, regardless of the location of fibrin: it can be extravascular deposits, which have been proposed as a marker of extravascular fibrinolysis correlated to the extent of lung injury (30). Fibrinolysis dysregulation in COVID-19 remains poorly understood. Several studies have shown that critically ill patients with COVID-19 display hypo-fibrinolysis (31, 32) or fibrinolysis shutdown associated with thrombosis, with the need for hemodialysis (33). Moreover, fibrinolytic shutdown should result in low D-dimer levels. Some studies have shown that COVID-19 patients have increased plasma thrombin levels and plasmin potential—in particular, high levels of plasmin-antiplasmin complex levels—compared with healthy donors and sepsis patients (34); nonetheless, *in vitro* analysis shows a hypofibrinolytic profile (31, 33) with an impaired response to r-tPA (32). Further studies are needed to better identify the role of fibrinolysis dysregulation in micro- and macrothrombosis and the origins of high D-dimer levels during COVID-19. Overall, it appears that FM detection could be a biomarker of worsening COVID-19 and could help in clinical characterization and management, regardless of the D-dimer level. Furthermore, FM quantification in plasma is now well standardized and is available on automated coagulometers with external quality controls.

Our study has some limitations: (i) the issue of missing data was addressed by imputation using a linear interpolation from observed values with an approximation function of the *stats* package of R software, (ii) the course of FM plasma levels could be influenced by DIC during ICU stay, and (iii) the course of FM plasma levels could be influenced by the different anticoagulation regimens.

All in all, the findings indicate that positive FM detected through iterative FM measurement during the first 9 days of hospitalization was associated with ICU referral and in-hospital mortality among COVID-19 patients. Hence, monitoring of FM during hospitalization could be an important tool for evaluating disease progression added to D-dimer. The predictive value should be confirmed in large multicentric studies that assess the association between routine measurement of FM levels and markers of thromboinflammation and endotheliopathy.

## Data Availability

The raw data supporting the conclusions of this article will be made available by the authors without undue reservation.
